# Comment on Keeler et al. Ketamine as a Treatment for Anorexia Nervosa: A Narrative Review. *Nutrients* 2021, *13*, 4158

**DOI:** 10.3390/nu14102118

**Published:** 2022-05-19

**Authors:** Maria Skokou

**Affiliations:** Department of Psychiatry, University Hospital of Patras, 26504 Patras, Greece; mskokou@upatras.gr; Tel.: +30-6977-906161 or +30-2613-603241

Anorexia Nervosa (AN) represents a difficult therapeutic challenge, with up to 4% prevalence among females and increasing incidence among youth [1]. Approved pharmaceutical treatments are virtually absent, and emerging treatments [2] have not been translated in therapeutic options, setting the scene for clinicians’ frustration [3]. Testing novel treatments, approved for frequent comorbidities of AN—for example depression—would seem reasonable, expecting that they might be effective on symptoms of AN.

Keeler et al. consider the case of using ketamine, currently approved for treatment- resistant depression, as a therapeutic option for severe, enduring AN, and describe the glutamergic and neuroplastic effects of the drug [4]. Taking this line of thought further, one could also consider the possible trial of other glutamatergic agents as effective for eating disorders, which could be lamotrigine, or vortioxetine. 

Lamotrigine is a phenyltriazine thought to reduce excess glutamate release, as a result of blocking voltage-sensitive sodium channels, and subsequent influx of sodium ions [5]. Further, it has antiaspartate, antikindling, neuroprotective and procognitive effects, and exerts weak 5-HT3 antagonism [6]. Being a first-line agent for acute bipolar depression and depressive relapse prophylaxis, it is also used for treatment resistant schizophrenia, treatment resistant OCD, unipolar depression, PTSD, depersonalization disorder, affective dysregulation and behavioral dyscontrol, all of which are frequently encountered in the context of AN [6]. Efficacy is coupled with a favorable side effect profile, except for serious rash; a careful, slow titration can mitigate this risk.

Vortioxetine is a novel antidepressant drug, exhibiting serotonin transporter inhibition, 5-HT-1A agonism, 5-HT-1B partial agonism, and 5-HT-1D, 5-HT-3, 5-HT7 antagonism. The blockade of 5-HT-3 receptors on GABA interneurons is responsible for the increase of glutamate in the prefrontal cortex and hippocampus, thus enhancing neuroplasticity and ultimately showing procognitive and antidepressant actions [7]. Its unique pharmacodynamic profile is shown by its superior efficacy regarding cognitive symptoms—attention, memory, processing speed and executive deficits—as well as generally benign adverse effects [8]. Particularly, apart from treating comorbid depression, promoting cognitive flexibility with vortioxetine could be of substantial relevance for the fixed distorted self-image, calorie intake and weight preoccupations of anorexic patients.

To my knowledge, there are no studies testing the efficacy of lamotrigine or vortioxetine for eating disorders except for scarce reports of lamotrigine [9,10]. The advantages of both drugs are their neuroplastic and procognitive actions, along with excellent tolerance, at a lower cost than intranasal ketamine, which would have to be used in a hospital or specialized setting. Still, the hypothetical efficacy of all remains to be tested, either each alone or in combination. In the end, for a potentially lethal, disabling, chronic disorder with practically no available pharmaceutical treatments, such as severe and enduring AN, we seem to need all the help we can get.
